# Upper gastro-intestinal bleeding — Rare presentation of renal cell carcinoma

**DOI:** 10.4103/0974-7796.68864

**Published:** 2010

**Authors:** Punit Tiwari, Astha Tiwari, Mukesh Vijay, Suresh Kumar, A. K. Kundu

**Affiliations:** Department of Urology, IPGMER and SSKM Hospital, Kolkata-20, West Bengal, India

**Keywords:** Metastatic renal cell carcinoma, stomach metastasis, upper gastrointestinal bleeding

## Abstract

Renal cell carcinoma (RCC) constitutes 2-3% of all adult malignancies and often diagnosed incidentally. Classical tried of RCC now rarely seen, it behaves unpredictably and having diverge range of clinical manifestation including paraneoplastic syndromes. Upper gastrointestinal (GI) bleeding due to stomach metastasis of RCC is uncommon and to the best of our knowledge, only few cases are reported in world literature and most of them were diagnosed during follow-up after complete treatment of RCC but in our case, it was the primary manifestation of disease. Our case also demonstrates the importance of imaging in undiagnosed cases of upper GI bleeding.

## INTRODUCTION

Renal cell carcinoma (RCC) accounts for 2–3% of all adult malignant neoplasm[1] and presents with different symptoms.[1] Because of liberal use of non-invasive imaging for nonspecific symptom, more than 50% of RCCs are now detected incidentally.[1] RCC may present with various symptoms due to local growth, metastasis, haemorrhage and sometimes paraneoplastic syndrome. Classical tried of flank pain, palpable abdominal mass and gross hematuria is now rarely seen. Approximately one third of the patients having metastasis at the time of initial diagnosis of RCC and accordingly presentation vary.[1] Upper gastrointestinal bleeding from stomach metastasis due to RCC is rare and under-recognized manifestation. We are reporting this rare case to highlight the importance of high index of suspicion and maintaining vigilance and liberal use of imaging in cases of undiagnosed upper GI bleeding. As the presenting symptom of a primary renal cell carcinoma, GI is described rarely in the literature. Bleeding is more commonly seen in patients as the first symptom of metastatic disease in patients who have previously undergone nephrectomy for RCC.

## CASE REPORT

**Figure 1:**
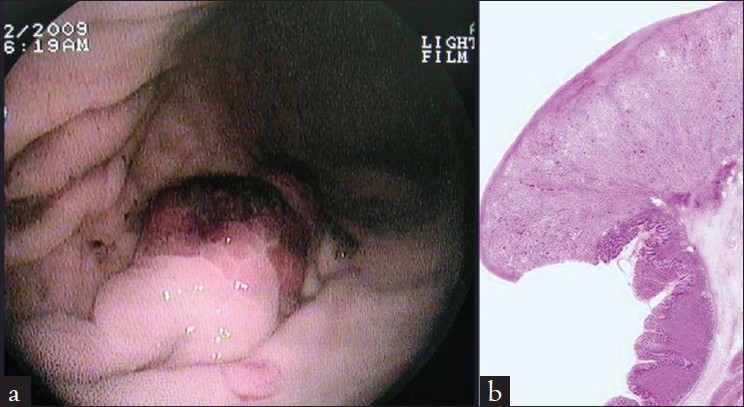
3–5 cm vascular polypoid mass was noted in the antrum of the stomach which was biopsied. (b) On histopathologic examination, the masses were confined to the mucosa and submucosa and consisted of sheets and nests of large, polygonal, clear cells, with distinct cell borders

**Figure 2:**
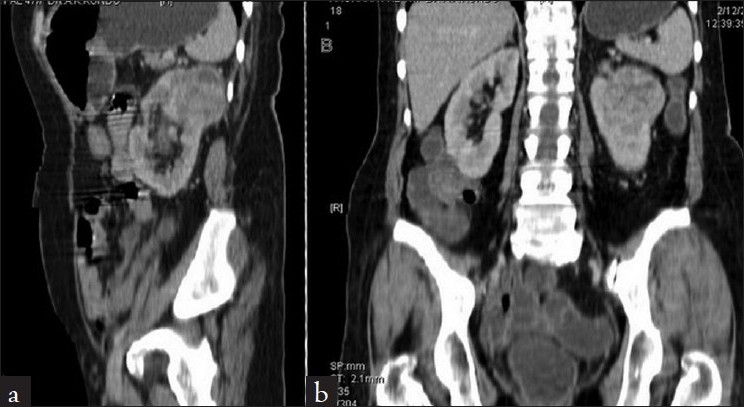
CECT abdoman showing contrast enhancing renal tumor involving upper pole of left kidney

A 58-year-old female presented to the emergency room with two-week history of melena, hemetemesis, generalized fatigue, and dizziness. She denied recent use of over-the-counter or nonsteroidal anti-inflammatory medications. Physical examination revealed a cachectic pale look. Blood pressure was 80/60 mm Hg, heart rate was 120 beats/min Abdominal examination revealed mild epigastric tenderness to deep palpation. There was no rebound tenderness or guarding or palpable masses. Laboratory tests revealed hemoglobin of 5.8 g/dl with microcytic picture, hematocrit of 16.5%, blood urea nitrogen of 24 mg/dl, creatinine of 0.8 mg/dl. Urinalysis did not reveal any red blood cells. White blood count, platelets, and coagulation studies were all normal. After initial resuscitation and blood transfusion, an emergent esophagogastroduodenoscopy was performed. A 3-5cm vascular polypoid mass was noted in the antrum of the stomach [Figure 1a], which was biopsied. No other lesions were noted in the oesophagus and the duodenum. Histology from the stomach mass revealed small, vacuolated, clear cells [Figure 1a]. Subsequent computed tomography (CT) of the abdomen revealed a well-defined mass in the superior pole of the left kidney [Figure 2a and b]. Her chest X-ray shows multiple pulmonary nodules, suggestive of primary renal cancer with metastatic disease. She underwent subtotal gastrectomy and with Roux-en-Y gastrojejunal reconstruction to prevent further episodes of re-bleeding. Because of the extensive metastatic disease, palliative therapy options were discussed with the patient but she declined any further interventions and died because of her disease two months later.

## DISCUSSION

RCC is one of the most lethal of the urologic cancers.[1] It arises from the proximal tubular epithelium of the kidney and has male preponderance (M:F 3:2). Mean age at presentation is 50-70 years. Most of the cases are sporadic and near about 4% are familial.[1] There are multiple risk factors includes increased age, smoking, obesity, long-term dialysis, exposure to asbestos, petroleum products and cadmium, and several genetic syndromes including familial clear cell carcinoma, von Hippel-Lindau syndrome, and tuberous sclerosis. Spread in RCC is lymphatic, haematogenous, transcoelomic, or by direct invasion.[1] Usual sites of metastasis from RCC are lung (75%), soft tissue (36%), bone (20%), liver (18%), cutaneous sites (8%) and central nervous system (8%).[2] Although RCC has a tendency to metastasize to unusual sites, cases of metastatic RCC to the stomach are very rare. The most common primary tumors metastasize to stomach are melanoma, carcinoma of the lung, breast and esophagus.[3] The tumors generally metastasize by hematogenous route and result in submucosal masses, as in this case. In a large series consisting of 23,019 autopsies, Davis and Zollinger found 67 metastases to the stomach from primary tumors outside the gastrointestinal tract none of them from the kidney.[4] Higgins reported 64 metastases in the stomach out of 31,541 examined autopsies. In this series also, none was from the kidney.[5] Gastrointestinal bleeding as a presenting symptoms of primary RCC rarely described in literature.[6] Bleeding is more commonly presenting feature of known patient of metastatic RCC or as a recurrence many years after nephrectomy for RCC.[7] Here we present a rare case of RCC whose primary manifestation of disease was with symptoms of upper gastrointestinal bleeding related to gastric metastases.

Gastric metastasis usually starts as a submucosal lesion, which encroaches onto the mucosa and becomes ulcerated. These lesions may be single or multiple and is grossly polypoid or plaque like.[3] Metastatic tumors can be distinguished from gastric carcinoma based on the absence of cellular atypia in the gastric glandular structures, which may appear compressed by the metastatic tumor. The most common presenting symptom is abdominal pain but nausea, hemetemesis, melena and gastrointestinal bleeding can be present. Gastrointestinal bleeding is mainly because of acid erosion of the metastatic lesion. Gastrointestinal bleeding as the presenting symptom of a primary renal cell carcinoma is described rarely in the literature.[6] Bleeding is more commonly encountered in patients already known to have metastatic disease, or as the first symptom of metastatic disease in patients who have previously undergone nephrectomy for RCC.[7] Here we present a rare case of RCC whose primary manifestation of disease was with symptoms of upper gastrointestinal bleeding. Diagnosis was confirmed by upper gastrointestinal endoscopy and histopathological examination of a biopsy. Surgical excision of gastric metastasis is essential as these lesions may bleed again after endoscopic coagulation treatment. This unique metastasis should be treated as a new tumor in stomach with prompt surgical excision. This often results in a significant survival prolongation with a good quality of life if the metastasis was solitary.[8] In the present case, though multiple pulmonary metastasis were present, surgery had to be performed because of the high risk of rebleeding. Palliative nephrectomy may alleviate local symptoms and can be undertaken in selected cases, but should be weighed against the burden of surgical morbidity and mortality. Outcome in metastatic RCC is generally poor, with one-year survival rate less then 50% and five-year survival rate of 5–30%.[1] Although evidence for the role of metastasectomy in RCC having multiple metastasis is lacking,[9] surgery (by subtotal gastrectomy and with Roux-en-Y gastrojejunal reconstruction) should be strongly considered in especially patients with git bleeding to prevent rebleeding and related complication.
